# Achieving optimal response at 12 months is associated with a better health-related quality of life in patients with chronic myeloid leukemia: a prospective, longitudinal, single center study

**DOI:** 10.1186/s12885-018-4699-5

**Published:** 2018-08-03

**Authors:** Lu Yu, Haibo Wang, Darko Milijkovic, Xiaojun Huang, Qian Jiang

**Affiliations:** 1 Peking University People’s Hospital, Peking University Institute of Hematology, No. 11 Xizhimen South Street, Beijing, 100044 China; 2 Peking University Clinical Research Institute, Beijing, China; 3 Novartis Pharma AG, Basel, Switzerland; 4 Collaborative Innovation Center of Hematology, Soochow University, Suzhou, China

**Keywords:** Chronic myeloid leukemia, CML, HRQoL, Nilotinib, Imatinib

## Abstract

**Background:**

To assess the relationship between responses within 1 year and health-related quality of life (HRQoL) outcomes by exploring profiles of patients with CML-CP who were treated with front-line imatinib or nilotinib.

**Methods:**

A prospective, longitudinal, single-center study was conducted to assess the response to treatment with imatinib or nilotinib and the HRQoL profile of patients who were newly diagnosed with CML in chronic phase enrolled in the ENESTchina study.

**Results:**

Fifty-nine patients were randomized to receive imatinib (*n* = 31) or nilotinib (*n* = 28). With a median follow-up of 5 years, there was no difference in HRQoL profile observed between patients receiving imatinib and nilotinib. Achieving optimal response at 12 months was associated with better role limitations due to physical health problems (RP; *P* = 0.0019) and emotional problems (RE; *P* = 0.0110) and was the sole factor associated with significantly improving physical component summary over time (PCS; *P* = 0.0160). Achieving optimal response at 6 months had high probability of better physical functioning (PF; *P* = 0.0674), better social functioning (SF; *P* = 0.0571), and reduced role limitations due to emotional problems (RE; *P* = 0.0916). In addition, factors including age < 40 years, female gender, and higher level of education were also associated with better HRQoL subscale scores. However, optimal response at 3 months had no impact on HRQoL profile. The proportions of patients with failure-free survival and PFS at 5 years were significantly higher among patients who achieved optimal response at 3, 6, or 12 months than among those who did not achieve optimal response (warning or failure), and the OS rate at 5 years was significantly higher among those who achieved optimal response at 12 months. In a multivariate analysis, treatment received (nilotinib vs imatinib) was identified as an independent factor for the achievement of optimal response at both 6 months (OR, 3.9; 95% CI, 1.0–14.9) and 12 months (OR, 5.6; 95% CI, 1.7–17.9).

**Conclusions:**

Achieving optimal response at 12 months was not only associated with longer OS and reduced treatment failure rates and disease progression but also better HRQoL in newly diagnosed patients with CML-CP receiving front-line tyrosine kinase inhibitor treatment.

**Trial registration:**

Chinese Clinical Trial Registry (http://www.chictr.org.cn): ChiCTR-OCH-11001699.

## Background

The introduction of imatinib in the year 2001 dramatically changed the treatment paradigm for patients with Philadelphia chromosome-positive (Ph+) chronic myeloid leukemia (CML) [1]. A number of studies revealed that imatinib as a first-line therapy induced a higher probability of achieving cytogenetic and molecular responses compared with previous therapies in CML patients in the chronic phase (CML-CP) [2, 3]. The 10-year progression-free survival (PFS) and overall survival (OS) rates were shown to be > 80% with imatinib [4]. In the past decade, second-generation tyrosine kinase inhibitors (TKIs) such as nilotinib and dasatinib further contributed to the remarkable improvement in clinical efficacy in terms of major molecular response (MMR, *BCR-ABL*^IS^ ≤ 0.1%) and molecular response 4.5 (MR4.5, *BCR-ABL*^IS^ ≤ 0.0032%) rates than imatinib as observed in the ENESTnd [5], DASISION [6], and ENESTchina [7] trials, despite no difference in the survival benefit compared with imatinib.

With the advent of TKIs, the life expectancy of patients with CML-CP is expected to be as normal as that of the general population [8]. Improvement in survival associated with treatment, necessitates the need for better understanding on the health-related quality of life (HRQoL) profile in these patients and is now recognized as an important component in the management of CML. Several studies have shown that imatinib significantly improves HRQoL in patients with CML-CP compared with hydroxyurea or interferon [9, 10]; younger aged patients and female patients had lower HRQoL scores than the general population. Furthermore, increasing age, lower level of education, more co-morbidities, advanced phase of CML, low-grade adverse events, and high out-of-pocket expenses for TKI therapy were significantly associated with an impaired HRQoL profile [9–17]. Decreased HRQoL may be associated with poor adherence to TKI therapy, which is a key factor contributing to treatment failure and unfavorable prognosis in patients with CML.

Several landmark studies have proved that early responses to TKI therapy are important milestones of survival [18–20], and patients treated with second-generation TKIs achieve a faster response than those treated with imatinib. Estimated PFS rates at 4 years in patients receiving nilotinib 300 mg twice daily who achieved early molecular response (EMR; *BCR-ABL*^IS^ ≤ 10% at 3 months) and who failed to achieve EMR were 95.2 and 82.9% (*P* = .0061), respectively, and estimated OS rates at 4 years were 96.7 and 86.7% (*P* = 0.0116), respectively [18]. Therefore, the question whether relatively early responses with TKI therapy could influence HRQoL outcomes in patients with CML-CP during long-term treatment remains unanswered. Most of the studies on HRQoL in patients with CML receiving TKI therapy are cross-sectional [14, 21] and longitudinal studies with long-term follow-ups, with a limited number of such studies in Asian patients are rare.

The primary objective of the current longitudinal single-center study was to prospectively explore patient-reported HRQoL profiles to identify demographic and clinical variables and treatment responses within 1 year with respect to HRQoL subscales in patients with CML-CP enrolled in the Evaluating Nilotinib Efficacy and Safety in Clinical Trials-China (ENESTchina) study. The secondary objective of the study was to compare the clinical efficacy of nilotinib and imatinib.

## Methods

### Patients

The current study was a part of the ENESTchina study of nilotinib vs imatinib [7] conducted at Peking University People’s Hospital. Newly diagnosed patients with CML-CP enrolled in ENESTchina were included.

Patients were treated, monitored, and followed according to the protocol of ENESTchina, as described previously [7]. All patients were diagnosed within 6 months of study entry, were aged > 18 years, and had an Eastern Cooperative Oncology Group performance status of 0–2. Patients were randomized to receive imatinib 400 mg once daily or nilotinib 300 mg twice daily. HRQoL was evaluated in these patients at baseline, every 3 months in the first 2 years, and then every 6 months over the next 3 years from the beginning of the study. Informed consent was obtained from each patient before screening for both treatment and evaluation of HRQoL. The study protocol was approved by the ethics committee of Peking University People’s Hospital, and the study is registered in the Chinese Clinical Trial Registry (http://www.chictr.org.cn) as # ChiCTR-OCH-11001699. The follow-up period defined as the time between treatment initiation and either premature withdrawal from the study (due to death or other reasons) or the end of the study, was 5 years.

### Assessment of response and outcome

Response to first-line TKI therapy and outcome definitions were previously derived from the 2009 European LeukemiaNet (ELN) criteria for the management of CML [22] when the HRQoL study was designed in June 2011, and were re-assessed according to the 2013 version [23] at the end of the study evaluation time in July 2016. Complete hematological response (CHR) was defined as white blood cell (WBC) count < 10 × 10^9/L, platelet count < 450 × 10^9/L, basophils < 5%, no blasts and promyelocytes in peripheral blood, myelocytes plus metamyelocytes < 5% in peripheral blood, and no evidence of extramedullary involvement at any assessment and confirmed by another assessment at least after 4 weeks.

Molecular responses were assessed by real-time quantitative reverse transcriptase polymerase chain reaction (RQ-PCR) and standardized to the International Scale (IS). Molecular responses were assessed at baseline, every 3 months for 3 years, and at the end of the study or on early discontinuation. Standard bone marrow cytogenetic assessments (> 20 metaphases) were performed at baseline and every 3 months thereafter until complete cytogenetic response (CCyR; defined as 0% Ph + metaphases by standard cytogenetics) was reached.

Definitions of warning and treatment failure were derived from the 2013 ELN criteria [23]. Warning was defined as *BCR-ABL1* > 10% and/or no partial cytogenetic response (PCyR; Ph + 36–95%) at 3 months, *BCR-ABL1* 1–10% and/or PCyR at 6 months, *BCR-ABL1* > 0.1–1% at 12 months, clonal chromosome abnormalities thereafter and at any time. Failure was defined as no CHR and/or no PCyR at 3 months, *BCR-ABL1* > 10% and/or PCyR at 6 months, *BCR-ABL1* > 1% and/or no CCyR at 12 months, loss of response (CHR, CCyR, or confirmed loss of MMR), or development of clonal chromosomal abnormalities or *BCR-ABL1* mutations after 12 months and thereafter at any time.

Failure-free survival (FFS) was defined as the time between treatment initiation and the appearance of treatment failure, not including discontinuation for toxicities. PFS was defined as the time between treatment initiation and progression to accelerated phase (AP), blast phase (BP), or death. OS was defined as the time between treatment initiation and death from any cause.

### Assessment of HRQoL

HRQoL was measured by the Medical Outcomes Study 36-item short-form health survey (SF-36) [24] in Chinese at baseline, every 3 months until 2 years, and every 6 months thereafter until 3 years or at the last outpatient visit during the study. Patients were regularly followed up as required by the protocol of ENESTchina and were asked to complete SF-36 questionnaires on paper using a pencil at each outpatient visit at Peking University People’s Hospital during the chronic phase. If a patient progressed to AP or BP during the study, data from the questionnaire of the respective patient at that visit were excluded and the patient was not followed up for HRQoL. The SF-36 is a well-established generic HRQoL measure with a questionnaire consisting of 36 items yielding 8 scales: physical functioning (PF), role limitation due to physical health problems (RP), bodily pain (BP), general health perceptions (GH), vitality (VT), social functioning (SF), role limitations due to emotional problems (RE), and mental health (MH) [24]. The 8 subscales are grouped to form 2 summary measures: the physical component summary (PCS) and the mental component summary (MCS). Higher scores represent better health outcomes.

### Statistical analysis

The median and range were provided for continuous variables, and percentages were provided for categorical variables. The Pearson chi-squared test (for categorical variables) and Mann–Whitney U test (for continuous variables) were used to measure between-group differences in variables. Missing QoL data were imputed by repeating the score of the last observation. Univariate analyses were performed to identify variables potentially associated with patients’ early responses at 3, 6, and 12 months during TKI therapy. Variables associated at a level of *P* < 0.2 in the univariate analysis were selected for the binary logistic regression model. The log-rank test was used to assess statistical significance in the time-to-event analyses. Variables including responses at 3, 6, and 12 months and demographic and clinical characteristics associated with the longitudinal change in HRQoL were analyzed in a mixed-model approach to linear regression for repeated measurements. Factors with nominal *P* < 0.05 level were identified as being potentially predictive of the outcomes. All analyses were conducted using SPSS version 22.0 and SAS version 9.3.

## Results

### Patient characteristics

A total of 59 patients were randomized to receive either imatinib (*n* = 31) or nilotinib (*n* = 28) at Peking University People’s Hospital during June 2011 to July 2011. The median age of the population was 37 years (range, 18–74 years), and 35 patients (59.3%) were male. Twenty-four patients (40.7%) had a bachelor’s degree or higher. Twenty-eight patients (47.5%) had low-risk Sokal scores, 18 (30.5%) had intermediate-risk scores, and 13 (22.0%) had high-risk scores.

**Fig. 1:**
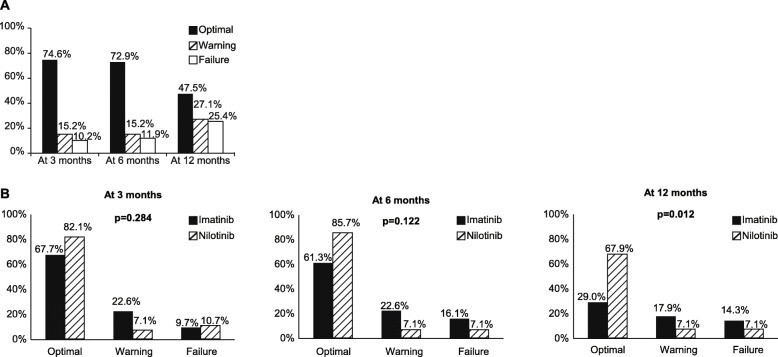
**a** Responses to treatment at 3, 6, and 12 months. **b** Responses to treatment at 3, 6, and 12 months by TKIs

All patients (100%) achieved CHR. With a median follow-up of 60 months (range, 9–61 months), 54 patients (91.5%) had achieved CCyR, 45 (76.3%) had achieved MMR, and 18 (30.5%) had achieved MR4.5. Based on the ELN criteria for response, 44 of 59 patients (74.6%) achieved optimal response at 3 months, 43 of 59 (72.9%) achieved optimal response at 6 months, and 28 of 58 (48.3%) achieved optimal response at 12 months (Fig. 1a). Patients treated with nilotinib had a higher probability of achieving optimal response at 12 months than those treated with imatinib (67.9% vs 29.0%; Fig. 1b).

### HRQoL

**Fig. 2:**
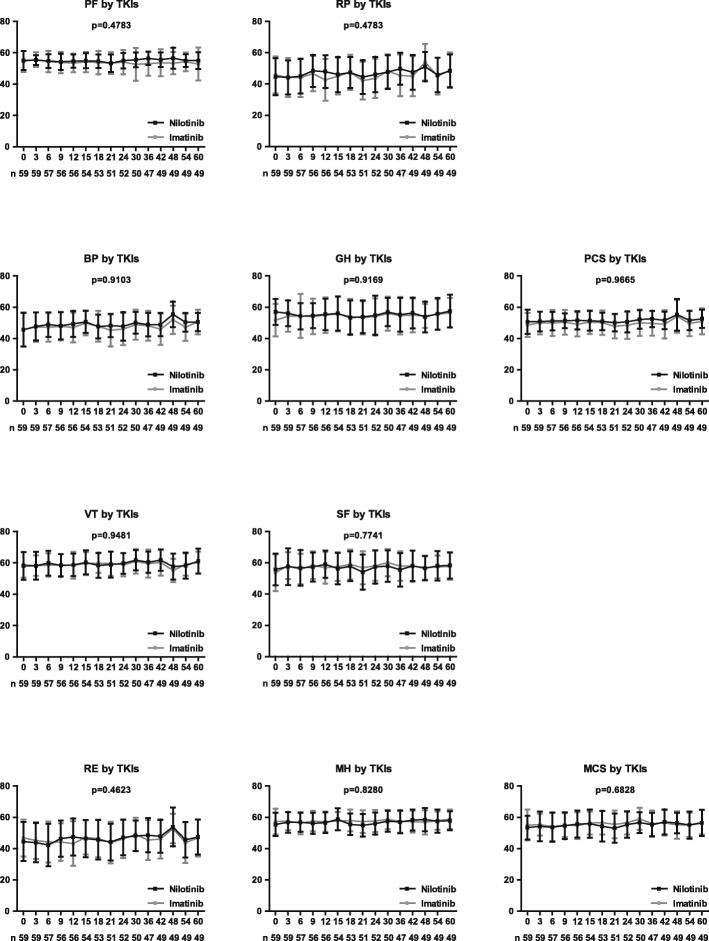
Health-related quality of life profile by TKIs

Marked differences were noted on some HRQoL subscales by demographic or clinical characteristics at baseline (Table 1), including SF by gender, MH and MCS by level of education, and GH by TKI used. However, each subscale score of HRQoL at baseline between patients achieving optimal response or not at 3, 6, or 12 months during TKI therapy was similar. During the 5-year follow-up period, there was no difference on each subscale score of the HRQoL profile, PCS scores, and MCS scores between patients receiving nilotinib and those receiving imatinib (Fig. 2).

**Table 1:** Subscales of health-related quality of life by patients’ characteristics at baseline and responses to TKI therapy within 1 year

Variable	No (%)	PF		RP		BP		GH		PCS		VT		SF		RE		MH		MCS	
		Median	*P*	Median	*P*	Median	*P*	Median	*P*	Median	*P*	median	*P*	Median	*P*	Median	*P*	Median	*P*	Meidan	*P*
Gender			0.61		0.75		0.58		0.44		0.84		0.36		***0.024***		0.18		0.91		0.087
Male	35 (59.3)	56.46		48.15		50.24		52.62		49.67		58.61		52.53		54.81		58.94		54.13	
Female	24 (40.7)	56.46		51.88		50.25		55.84		51.14		61.00		59.55		54.81		58.06		58.76	
Age			0.056		0.66		0.55		0.78		0.62		0.96		0.73		0.14		0.42		0.85
< 40 years	33 (55.9)	56.46		48.15		54.15		56.58		50.29		58.61		59.55		54.81		57.18		56.54	
≥ 40 years	26 (44.1)	53.94		51.88		46.34		53.86		50.31		58.61		52.53		49.67		58.94		55.40	
Education			0.36		0.85		0.40		0.54		0.64		0.19		0.66		0.14		***0.026***		***0.030***
≥ Bachelor degree	24 (40.7)	56.46		48.15		54.15		55.35		51.94		62.20		52.53		54.81		60.70		52.60	
< Bachelor degree	35 (59.3)	56.46		55.60		46.34		55.10		49.67		58.61		59.55		54.81		55.42		59.58	
TKI used			0.52		0.71		0.98		***0.042***		0.38		0.87		0.49		0.49		0.23		0.40
Imatinib	31 (52.5)	56.46		55.60		54.15		52.62		49.01		58.61		52.53		54.81		58.94		57.29	
Nilotinib	28 (47.5)	57.73		48.15		50.24		58.32		52.21		59.81		59.55		54.81		57.18		52.93	
Sokal risk			0.63		0.31		0.67		0.48		0.17		0.29		0.32		0.84		0.47		0.99
Low	28 (47.5)	56.46		48.15		48.29		54.60		48.35		58.61		52.53		54.81		58.94		56.07	
Intermediate / High	18 (30.5) /13 (22.0)	56.46		55.60		54.15		55.10		52.52		58.61		59.55		54.81		57.18		56.54	
Response at 3 months			0.35		0.26		0.30		0.36		0.20		0.71		0.94		0.41		0.83		0.80
Optimal	44 (74.6)	56.46		48.15		48.29		52.62		49.20		58.61		56.04		54.81		58.06		56.60	
Warning / Failure	9 (15.3) /6 (10.1)	58.99		55.60		54.15		59.06		52.28		58.61		52.53		54.81		58.94		55.22	
Response at 6 months			0.52		0.45		0.77		1.0		0.35		0.44		0.52		0.89		0.74		0.92
Optimal	43 (72.9)	56.46		48.15		54.15		55.10		49.38		58.61		52.53		54.81		58.94		55.46	
Warning / Failure	9 (15.3) /7 (11.9)	57.73		55.60		48.29		55.35		51.29		59.81		56.04		54.81		56.30		57.09	
Response at 12 months			0.65		0.85		0.49		0.92		0.29		0.43		0.91		0.37		0.64		0.40
Optimal	28 (48.3)	56.46		48.15		48.29		53.86		48.74		59.81		56.04		54.81		58.06		57.11	
Warning / Failure	16 (27.6) / 14 (24.1)	56.46		55.60		54.15		56.58		52.12		58.61		52.53		54.81		58.94		55.22	

*P* value in bold and italics are significant with *P* < 0.05

**Fig. 3:**
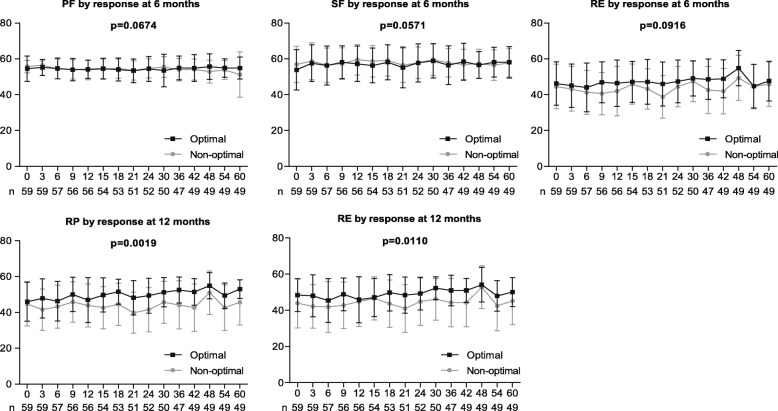
Health-related quality of life profile by treatment response at 6 and 12 months

**Fig. 4:**
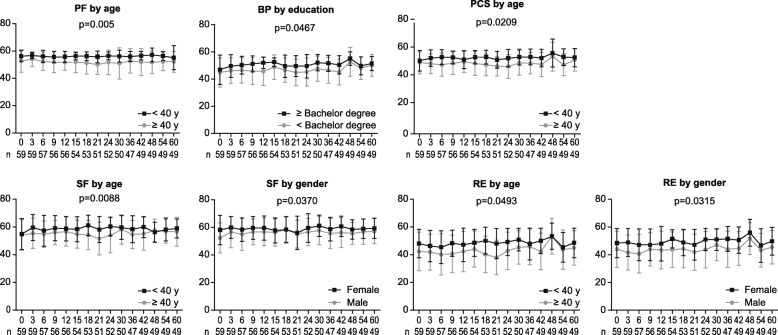
Health-related quality of life profile by demographic characteristics at baseline

Treatment responses at 3, 6, and 12 months; demographic and clinical characteristics at baseline; and TKIs used were assessed to identify the factors associated with a better HRQoL profile in patients receiving TKI therapy. Multivariate analyses showed that achievement of optimal response at 6 months was associated with a tendency of having high PF (*P* = .0674), SF (*P* = 0.0571), and RE (*P* = 0.0916) scores, while achieving optimal response at 12 months was associated with markedly higher RP (*P* = 0.0019) and RE (*P* = 0.0110) scores (Fig. 3). In addition, age < 40 years was associated with better PF (*P* = 0.0005), PCS (*P* = 0.0209), SF (*P* = 0.0008), and RE (*P* = 0.0493) scores; female gender was associated with better SF (*P* = 0.0370) and RE (*P* = 0.0315) scores; and a higher level of education was associated with better BP (*P* = 0.0467) (Fig. 4). Response at 3 months and the TKI used (imatinib or nilotinib) did not show any impact on the HRQoL outcomes during TKI therapy.

**Fig. 5:**
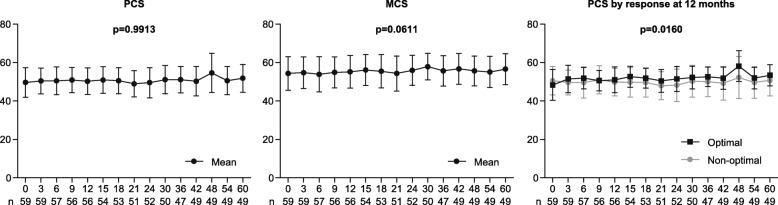
Physical component summary, mental component summary and physical component summary by treatment response at 12 months over time

Furthermore, we assessed the factors including treatment responses, patient characteristics, TKI used, and duration of therapy associated with the longitudinal change in the HRQoL profile in patients on TKI therapy. Multivariate analyses showed that PCS scores were constant throughout the treatment (*P* = 0.9913), while MCS scores showed a tendency toward gradual increase (*P* = 0.0611) with continuation of treatment; however, achieving optimal response at 12 months was the sole factor associated with a significant improvement in PCS scores over time (*P* = .0160; Fig. 5).

### Progression and survival

With a 5-year follow-up, 7 patients progressed to AP or BP, 4 died because of disease progression, and 3 dropped out of the study because of unsatisfied response or adverse effects. The proportions of patients with FFS, PFS, and OS at 5 years were 69.5, 88.1, and 93.2%, respectively. The rates of FFS and PFS at 5 years were significantly higher in patients who achieved optimal response at each time point of 3, 6, or 12 months than those who did not achieve optimal (warning or failure) response. The 5-year FFS rates for patients who achieved optimal response vs non-optimal response were 84.1% vs 26.7% at 3 months, 90.7% vs 12.5% at 6 months, and 100% vs 41.9% at 12 months. The 5-year PFS rates for patients who achieved optimal response vs non-optimal response were 93.2% vs 73.3% at 3 months, 93% vs 75% at 6 months, and 100% vs 77.4% at 12 months.

**Fig. 6:**
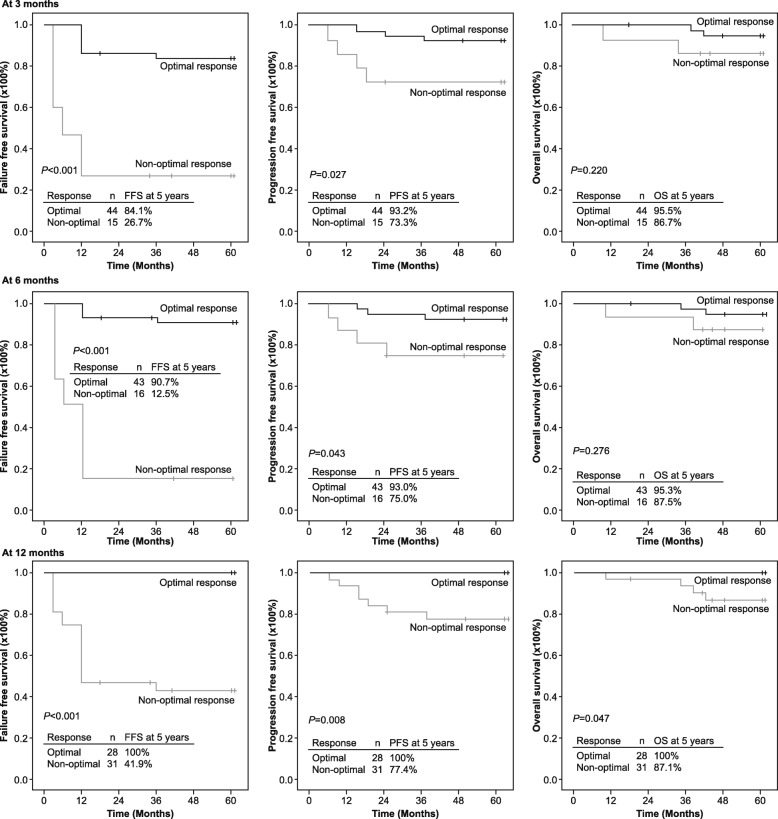
Failure-free survival, progression-free survival, and overall survival by treatment response at 3, 6, and 12 months

There was a slight difference in the rates of OS at 5 years between patients who had optimal response or non-optimal response at 3 months (95.5% vs. 86.7%) or 6 months (95.3% vs. 87.5%). However, the OS rate at 5 years was numerically higher in patients who achieved optimal response (100%) compared with patients with non-optimal response (87.1%) at 12 months (Fig. 6).

### Factors associated with achieving optimal responses

Variables including gender (male vs female), age (< 40 years vs ≥ 40 years), level of education (bachelor’s degree vs no bachelor’s degree), Sokal risk score (low vs intermediate and high), and TKI used (imatinib vs nilotinib) were assessed to identify the factors associated with achieving optimal response at 3, 6, and 12 months. Multivariate analyses showed that gender (female vs male: odds ratio [OR] = 5.2; 95% CI, 1.2–23.2) and Sokal risk score (low vs intermediate and high: OR = 4.7; 95% CI, 1.2–18.9) were associated with achieving optimal response at 3 months. Sokal risk score (low vs intermediate and high: OR = 3.9; 95% CI, 1.0–14.9) and treatment received (nilotinib vs imatinib: OR = 3.9; 95% CI, 1.0–14.9) were associated with achieving optimal response at 6 months, while only treatment received (nilotinib vs imatinib: OR = 5.6; 95% CI, 1.7–17.9) was associated with achieving optimal response at 12 months.

## Discussion

This prospective longitudinal study found that achieving optimal response at 12 months improved HRQoL including better RP and RE during TKI therapy in newly diagnosed patients with CML-CP treated with imatinib or nilotinib. Achieving optimal response at 12 months also significantly improved PCS over time with a 5-year follow-up. Moreover, early optimal response is associated with favorable long-term outcomes including low rates of treatment failure, disease progression, and deaths. The probability of achieving optimal response at 12 months was higher with nilotinib compared to imatinib.

Similar to previous reports [25–27], our study also revealed that patients with CML-CP on front-line TKI who did not achieve early optimal response at 3, 6, and 12 months had higher probabilities of treatment failure and disease progression to AP or BP. Trask et al. [13] reported that individuals with CML in the AP or BP had poorer HRQoL than those in the CP using functional assessment of cancer therapy-leukemia (FACT-Leu). However, in the current study, all SF-36 patient-reported outcome (PRO) questionnaires (the most well-established generic HRQoL measure) were collected from patients only in the CML-CP and not in CML-AP. There are studies conducted in the recent past that have assessed the HRQoL using both narrative and quantitative techniques among patients with CML [10, 12, 13, 15, 28, 29]; however, the use of standardized PRO tools simplifies the data analysis and interpretation.

Our findings showed that achieving optimal response at 12 months was associated with more favorable HRQoL. Although optimal response at 3 months was associated with higher FFS and PFS rates, it had no impact on the HRQoL profile of patients. It is possible that achieving optimal response at 6 months was associated with a tendency for a state of well-being compared with those with a non-optimal response, as indicated in the small number of patients studied. Armstrong et al. reported that adalimumab treatment resulted in a statistically significant and clinically relevant reduction in disease severity that was associated with QoL improvement in patients with psoriasis compared with placebo [30]. Hess et al. reported that patients with mantle cell lymphoma achieving a partial or better clinical response showed an improvement in FACT-Lym total scores [31]. Similarly, our results reflect that effective TKI treatment leads to an improvement in physical and mental health in patients with CML, suggesting that TKI therapy responses within 1 year can possibly predict both future treatment outcomes and physical and mental well-being of patients with CML-CP. The SPIRIT2 trial, a comparison study between imatinib and dasatinib in newly diagnosed patients with CML, showed no significant difference in HRQoL with generic and cancer-specific instruments [32]. In the current study, although the HRQoL outcome did not vary with the use of different TKIs (imatinib or nilotinib), the use of nilotinib was identified as an independent factor associated with achieving optimal response at 6 and 12 months. Therefore, in comparison to imatinib, nilotinib-induced response may indirectly benefit HRQoL outcomes in patients with CML-CP.

As reported earlier [16, 17, 28], younger patients (age < 40 years) and patients with a bachelor’s degree or higher had better physical and/or mental health. Previous studies have reported that male patients had better physical and mental health than female patients [16, 21, 28]. In contrast, female gender was associated with better SF and RE in the present study; however, higher SF scores in female patients at baseline may have contributed to their better SF during therapy.

There were some limitations in the current study. The small sample size was the primary limitation to detect the true effect of variables (PF, SF, and RE) on achieving response. A larger sample size would have supported interpretation of the results by means of achieving statistical significance rather than a simple estimation of tendency between optimal response at 6 months and PF, SF, and RE. Further, the SF-36 questionnaire may not be sensitive enough to detect QoL changes in the CML population because it is not a cancer-specific instrument. In addition, the study population was relatively young and in good health due to stringent criteria of excluding patients with co-morbid conditions, organ impairment, or severe or uncontrolled medical conditions. These findings show that better responses at 12 months (or 6 months) were associated with better HRQoL, and the results should be confirmed in future studies with a larger sample size including all age groups.

Achieving an early optimal response associated with nilotinib may not only help patients in attempting the treatment-free remission (TFR), but also to have better HRQoL. Nilotinib is the first and only TKI to include information on stopping the therapy in patients with Ph + CML-CP as approved by both the European Commission [33] and the United States [34]. When Hochhaus et al. evaluated the impact of TFR on patient’s QoL prior to, during, or after TFR, no effect of stopping the treatment was observed; in addition, the reported levels of anxiety/depression were similar between during and after TFR [35].

## Conclusion

In conclusion, the use of TKI (imatinib or nilotinib) did not show any impact on HRQoL outcomes during TKI therapy. Each subscale score of HRQoL at baseline between patients achieving optimal response and those not achieving optimal response at 3, 6, or 12 months during TKI therapy was similar. Achieving optimal response by 12 months was not only associated with longer survival and lower rates of treatment failure and disease progression but also better HRQoL in newly diagnosed patients with CML-CP on front-line TKI. Further studies with larger sample sizes are required to confirm these findings.

## Abbreviations

AP: Accelerated phase
BP: Blast phase
CCyR: Complete cytogenetic response
CHR: Complete hematological response
CI: Confidence interval
CML: Chronic myeloid leukemia
CML-CP: Chronic myeloid leukemia in chronic phase
ELN: European LeukemiaNet
FFS: Failure-free survival
HRQoL: Health-related quality of life
MCS: Mental component summary
MMR: Major molecular response
MR4.5: Molecular response 4.5
OR: Odds ratio
OS: Overall survival
PCS: Physical component summary
PCyR: Partial cytogenetic response
PFS: Progression free survival
Ph +: Philadelphia chromosome-positive
SF-36: 36-item short-form health survey
TKI: Tyrosine kinase inhibitor
WBC: White blood cell
